# Speed Trends in Male Distance Running

**DOI:** 10.1371/journal.pone.0112978

**Published:** 2014-11-19

**Authors:** Timothy N. Kruse, Rickey E. Carter, Jordan K. Rosedahl, Michael J. Joyner

**Affiliations:** 1 The University of Washington School of Medicine, 1959 N. E. Pacific Street, Seattle, WA, 98195, United States of America; 2 Department of Health Sciences Research, Mayo Clinic, 200 First Street SW, Rochester, MN, 55905, United States of America; 3 Department of Anesthesiology, Mayo Clinic, 200 First Street SW, Rochester, MN, 55905, United States of America; University of Rome, Italy

## Abstract

The major cycling “Grand Tours” have shown an attenuation of performance over the last decade. This has been interpreted as circumstantial evidence that newer anti-doping strategies have reduced the use of performance-enhancing drugs. To examine this idea under more controlled conditions, speed trends for world class 5000 m, 10000 m, and marathon performances by men from 1980 to 2013 were analyzed. We obtained comprehensive records from the International Association of Athletics Federations, Association of Road Racing Statisticians, and the Track and Field All-time Performances database webpages. The top 40 performances for each event and year were selected for regression analysis. For the three distances, we noted cumulative performance improvements in the 1990s thru the mid-2000s. After the peak speed years of the mid 2000 s, there has been limited improvement in the 5000 m and 10,000 m and world records set during that time remain in place today, marking the longest period of time between new records since the early 1940s. By contrast marathon speed continues to increase and the world record has been lowered four times since 2007, including in 2013. While the speed trends for 5000 m and 10000 m track results parallel those seen in elite cycling, the marathon trends do not. We discuss a number of explanations other than improved anti-doping strategies that might account for these divergent findings.

## Introduction

The use of performance-enhancing drugs (doping) can be dated back to the ancient Olympics [1], [2]. Since then athletes have used a wide range of substances including red wine, caffeine, nitroglycerin, cocaine, opiates, amphetamines, growth hormone, blood transfusions, anabolic steroids, and erythropoietin (EPO) in an effort to gain a physiological advantage [3]. Because performance-enhancing drugs compromises the idealized principles of pure competition, the World Anti-Doping Agency (WADA) was created in 1999 [4]. Since the formation of WADA, it has developed widely applied policies and drug testing protocols (including regular out of competition testing) in an attempt to stop the apparently wide spread doping in elite sports competition [5].

If recent improvements in athletic performance have been driven by doping, then improved doping control might be reflected by a leveling off or declining performances in sports where doping is thought to be ubiquitous. In recent analyses of major cycling races including the Tour de France, Giro d’Italia, Vuelta A España, the average speed has been leveling off or declining [6], [7] since the introduction of improved techniques to detect use of exogenous EPO in 2005 [4]. However, the analysis of cycling is confounded by varying race distances, yearly changes in course, and weather. Endurance running eliminates many of these confounding factors. The tracks and courses are identical from year to year. For the two shorter distances, there are numerous competitive opportunities per year and at least some would likely have nearly ideal environmental conditions.

With this information as a background, we tested the hypothesis that elite distance running times would show a pattern of leveling in the middle 2000 s similar to that seen in cycling. Such a finding could be explained by improved doping control. We also discuss alternate explanations including that humans are reaching the biological limits of performance and the potential role technical innovations in training and equipment [8], [9]. Finally, any potential explanation might also be confounded by changes in the economic landscape associated with world class distance running.

## Materials and Methods

We obtained the top male performances from 1980–2013, by year, in major endurance running races (5000 m, 10000 m on the track; marathon on road courses) from the International Association of Athletics Federations (IAAF: http://www.iaaf.org/home), Association of Road Racing Statisticians (ARRS: http://www.arrs.net), and the Track and Field all-time Performances database websites (http://www.alltime-athletics.com/index.html) [10], [11], [12].

The 1980–2013 epoch was selected because besides new performance-enhancing drugs (PEDs), a case can be made that potentially transformative changes in training or equipment has not occurred. For example, by 1980 high volume and high intensity training had been widely adopted by top competitors for several decades and athletes from East Africa had been participating at the international level since the early 1960s. High quality synthetic tracks were also widely available by 1980 and carbohydrate loading was widely practiced in the marathon and it is unclear if technical changes in shoes have had a measureable impact on performance. While ideas about training have been refined the extent to which these have been uniformly adopted by elite athletes, especially the East Africans, is not known [13], [14], [15].

Beyond these training and globalization related factors, professionalism also emerged during the 1980s. We also restricted our analysis to men because women were not routinely permitted to participate in long distance racing until the 1970s and performances dropped dramatically in the early years of widespread competition by women. While the gap in world records has been steady since that time women still lag in competitive depth in many events [16], [17].

Finally, the first synthetic EPO (Epogen) was approved by the FDA in 1989 and within a few years it was clear that EPO can have profound effects on maximal oxygen transport (VO2 max) [18] in humans and was being used to enhance athletic performance by the early 1990s. Additionally, because EPO and related analogues are injectable the logistical challenges of traditional blood doping (autologous red cell transfusions) are eliminated [19].

The abstracted data consisted of the total number of performances below 2012’s Olympic A standard: 5000 m-13∶20, 10,000 m-27∶45, and also performances under 2∶10∶00 for the marathon (the Olympic A standard was 2∶15 in 2012, a time that equals an estimated 29.22 10,000 m [20]). Performances below these standards were considered ‘elite’. To study the speed tends more formally, the 40 fastest athlete performances (the fastest performance of the 40 fastest athletes) were recorded per year and event for regression modeling (described below). Age and country of residence at time of race were also abstracted.

Initial data summaries included the frequency tabulation elite performances by event and distance. To analyze the changes in speed trends over the study period, we used regression techniques consisting of quadratic splines (cubic B-splines with 3 equally spaced interior knots) against our years of interest using the top 40 athlete performances per year. These generalized regression models allowed for flexibility of estimating the change in performance over time while providing traditional measures of model fit (e.g., R-square value). It was hypothesized that different regression profiles would be observed between the top 40 finishing times and the fastest yearly performances, so the percentage difference in speed of the fastest performance relative to the 40th fastest time per year was also modeled using regression splines and locally weighted smoothers (LOESS). When reporting measures of model fit for the cubic B-splines regression models (i.e., change in speed as a function of year), the omnibus F test for the regression model and R-square were reported. The LOESS curves, which were used for illustrative purposes of changes in pacing based on the relative placing, were not summarized using traditional regression summaries such as R-square on account of their intended visual utilization.

Cubic B-spli ne regression analyses were conducted using The SAS System (v9.3, Cary, NC) using PROC ORTHOREG. LOESS smoothing was conducted using PROC SGPLOT using default parameters.

## Results

**Figure 1:**
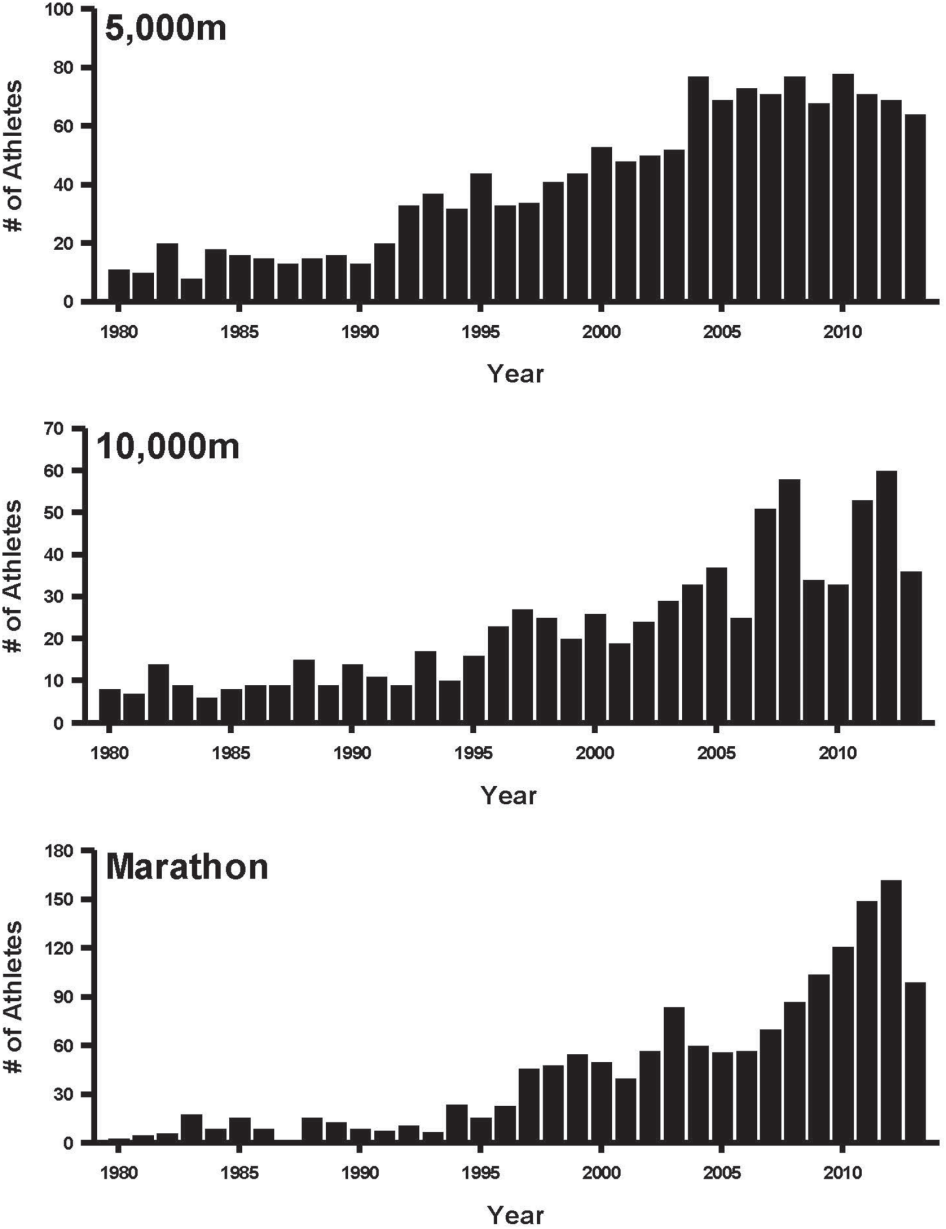
Total number of elite performances by year. Times under 13∶20 for the 5000 m, 27∶45 for the 10000 m, and 2∶10 for the marathon.

The number of performances below the 2012 Olympic A qualifying standard plus sub 2∶10∶00 for each distance increased over the 1980–2013 (Figure 1a–c). The world record for the 5000 m was set in 2004 while the 10000 m world record was set in 2005; these records stand today, which is the longest gap between world records since the 1940s. The number of performances below the 2012 Olympic A qualifying standard for the 5000 m and 10,000 m also appears to have leveled off since the middle 2000 s. Similarly the number of athletes breaking 2∶10∶00 for the marathon has also leveled off. 2∶10∶00 was chosen as a comparable standard for the marathon because this time is considered generally similar to or slightly slower than the A standards for shorter distances based on various empirical point tables, scoring systems and time conversion programs [20].

**Figure 2:**
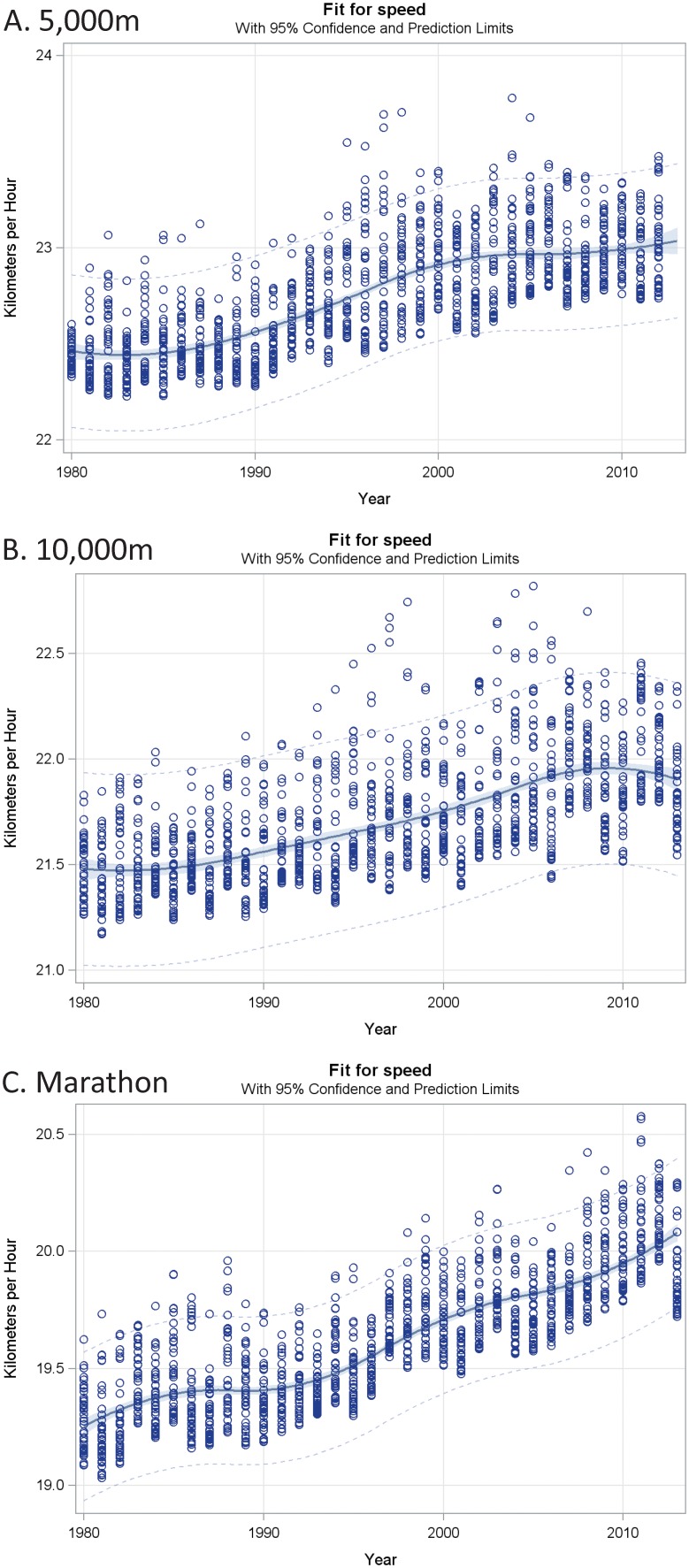
Top 40 athlete performances of the 5000 m, 10000 m, and marathon (circles). The solid line is a quadratic spline fit with three equally spaced knots (points of inflection).

All three regression splines presented in Figures 2a–c were statistically significant (p<0.0001 for each). Furthermore, year alone explained a large percentage of the variation in the speed trends (R-square: 53%, 37% and 69% for 5000 m, 10000 m and marathon, respectively). Consistent with these overall model estimates, the 5000 m and 10000 m had significant increases in speed during the 1990s whereas the marathon showed an increase over the entire three plus decades (Figures 2a–c). In particular, the 5000 m the speed trend levels off starting around 2000. The marathon and 10000 m times do not show this as a pronounced tendency.

**Figure 3:**
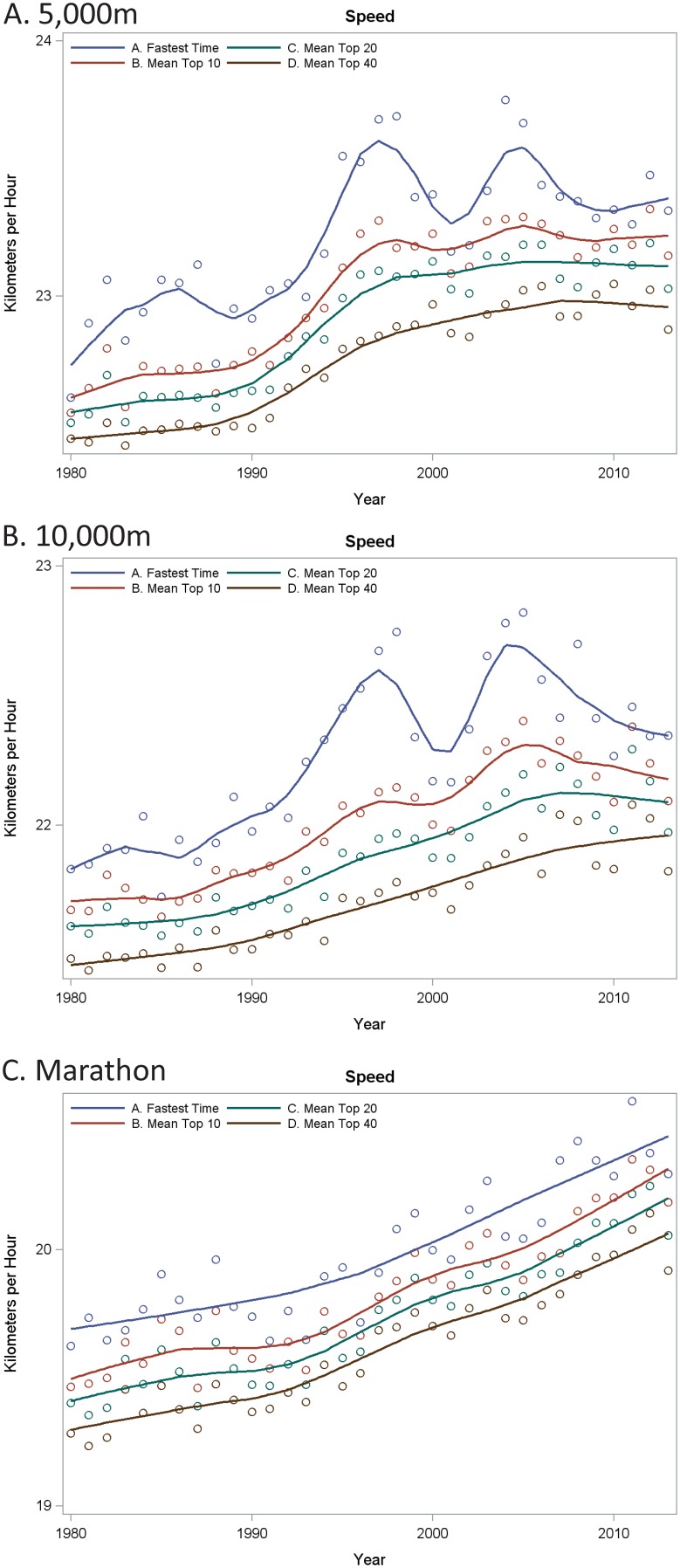
LOESS smoothers fit through the fastest yearly performance by year for the 5000 m (A), 10000 m (B) and marathon (C). In addition, the mean speed for the top 10, top 20 and top 40 athlete performances are also plotted for reference.

Figures 3a–c explores the speed trends using an alternative classification approach to provide additional insights into the temporal effects. The fastest performance of the year is plotted alone and summarized using a LOESS smoother. Speed trends of the mean top 10, mean top 20 and mean top 40 athlete performances are superimposed in these same figures. With the 5000 m and 10000 m, there is a pronounced ‘outlying’ effect of the top performance from 1995 to late 2010. The marathon, however, displays no attenuation of the increased speed over the epoch sampled and the relative speed of the fastest annual time does not appear to be an outlier (i.e., the figure lines appear as roughly parallel).

To better quantify the observations made from Figure 3, the percentage changes in speeds over time were examined and found to be consistent with the differential findings of the top performance vs. the 40^th^ fastest athlete performance. The degree to which the fastest times were relatively fast (compared to other years) was observed during the 2000s in the 5000 m and 10000 m distances. The regression spline analyses supported these findings that the fastest relative times for the 5000 m and 10000 m varied over the epoch (5000 m: p = 0.048, R-square = 32%; 10000 m: p = 0.0007, R-square = 51%). As illu strated in Figure 3c, trends for the marathon distance were not clearly identified in the data (p = 0.29, R-square = 19%).

## Discussion

The speed and performance trends for top 5000 m and 10000 m distance running performers on the track show a period of increased speed among the fastest runners to the mid-2000 s with an attenuation of speed in either all (5000 m) or the fastest performances (10000 m) after this period of time. For the marathon, all indices of speed show a nearly linear increase in speed with an increased number of elite performances over the three plus decades we sampled. We believe there are a number of possible explanations for these findings.

First, the findings for the 5000 m can be interpreted as consistent with the hypothesis that improved drug testing has limited the ability of elite athletes to manipulate their oxygen transport systems with EPO (or other techniques to improve oxygen transport during exercise) since the middle 2000 s. These observations are also broadly consistent with recent speed trends in elite cycling races [6], [7]. This interpretation can also be applied to the 10000 m results, but only when considering the fastest times. By contrast, the data for the marathon shows continued improvements in running speed during the same time period along with more total elite performances and world records. These observations challenge the idea that the speed leveling seen in the 5000 m on the track and in the so-called “Grand Tours” of cycling is due primarily to better drug testing and the reduced use of performance enhancing drugs.

A second possible interpretation is that world class performances are leveling off and reaching a physiological upper limit as has been postulated for equine and canine athletes [8], [9,], [21]. In the case of the marathon a number of empirical estimates and physiological modeling suggest the record is relatively slow in comparison to the 5,000 m and 10,000 m times and is merely catching up by comparison [20], [22], [23]. In this context, it is interesting to note that top speeds have not fallen for the shorter races but only leveled off.

The third element of any interpretation focuses on the changing financial incentives in professional distance running. Prize money for top marathon performances has increased. Specifically, in 1980 the highest total payout for any marathon was $50,000; just over two decades later the first million dollar race was run [24]. These incentives could be attracting a stronger pool of competitors to “move up” and focus on the marathon and forgo record setting attempts at shorter distances. This could lead to more competitive races among top runners at the major marathons. Second the highest profile marathon races are now being staged in a way designed for world record attempts that include the use of pacers. Along these lines, the use of pacers has been wide spread for races on the track for many years, and many top athletes have bonus plans and other financial incentives from sponsors that reward fast times at the shorter distances. There are a number strengths and limitations to this study. A major strength of our data set and analysis is that it includes standardized distances and courses with numerous competitive opportunities at the shorter two distances when environmental conditions are likely to be optimal. By contrast, a limitation to our analysis is that we have no idea if improved approaches to training or equipment (shoes and tracks) might have contributed to the trends we report. However, we favor the interpretation that the entire epoch we have analyzed has been relatively stable from a technical perspective. This includes widespread use of high volume and high intensity training, widespread availability of synthetic tracks, and adequate footwear. Additionally, while ideas about training have been refined it is not known if how uniformly these have been adopted by elite athletes, especially the East Africans [13], [14], [15]. This perspective contrasts to the major improvements in equipment for cycling that includes use of advanced materials and improved aerodynamic designs to construct faster bikes.

A final concern whenever the topic of doping is raised is discussed relates to what might be called the continuous “cat and mouse” game between those trying to enforce the rules with improved testing and those trying to circumvent them. This has engendered speculation that micro-doses of EPO can be titrated by athletes in a way to achieve high levels of performance and yet avoid a positive drug test [19], [25], [26]. There is also widespread speculation about the use less or undetectable compounds and so-called designer performance enhancing drugs. Advocates of these points of view have argued that while doping is considered widespread the number of positive tests in major competitions is quite low [5]. The counter argument is that the low number of positive results demonstrates that the testing is working and deterring doping. The lack of hard data on the true incidence of doping and how it has or has not been influenced by improved testing is unknown and a major limitation to any discussion on this topic. However, it is clear that anonymous questionnaire based surveys suggest the true incidence of doping it is much higher (14–39%) than ∼2% rate of positive tests suggests [27]. This is clearly an area of sports sociology that requires increased attention.

It should also be noted that the sociology surrounding the doping phenomenon along with the ongoing incentives to dope are complex. In this context, strategies beyond testing alone will be required to improve the efficacy of doping control. A comprehensive discussion of this complex topic is beyond the scope of our analysis, but there has been much thoughtful discussion of related topics [28], [29], [30], [31], [32], [33], [34].

## Conclusion

In summary, our analysis demonstrates that speed trends for elite distance running are divergent depending on distance and have event specific patterns. Thus, any generalizations about performances in world class competition providing evidence that drug testing is or is or is not “working” need to be viewed with caution. Further caution is required given the many caveats and potential factors that could explain our findings.
